# Global health: the importance of evidence-based medicine

**DOI:** 10.1186/1741-7015-11-223

**Published:** 2013-10-16

**Authors:** Gretchen L Birbeck, Charles S Wiysonge, Edward J Mills, Julio J Frenk, Xiao-Nong Zhou, Prabhat Jha

**Affiliations:** 1 University of Rochester Medical Center, School of Medicine and Dentistry, 601 Elmwood Ave, Box CU420694, Rochester, NY 14642, USA; 2 Centre for Evidence-based Health Care, Faculty of Medicine and Health Sciences, Stellenbosch University, Tygerberg Campus, Cape Town 7505, South Africa; 3 Faculty of Health Sciences, University of Ottawa, 25 University Private, Ottawa ON K1N 6N5, Canada; 4 Stanford Prevention Research Center, Stanford University, 291 Campus Drive, Stanford, CA 94305510, USA; 5 Harvard School of Public Health, Kresge Building, Room 1005, 677 Huntington Avenue, Boston, MA 02115, USA; 6 Chinese Center for Disease Control and Prevention, National Institute of Parasitic Diseases, 207 Rui Jin Er Road, Shanghai 200025, PR China; 7 Centre for Global Health Research, St. Michael’s Hospital, Dalla Lana School of Public Health, 30 Bond Street, Toronto M5B 1W8, Canada

**Keywords:** Global health, Policy, Evidence-based medicine, Research output, Research methodology, Poverty, Infectious diseases, Health coverage, Resource-limited settings

## Abstract

Global health is a varied field that comprises research, evaluation and policy that, by its definition, also occurs in disparate locations across the world. This forum article is introduced by our guest editor of the *Medicine for Global Health* article collection, Gretchen Birbeck. Here, experts based across different settings describe their personal experiences of global health, discussing how evidence-based medicine in resource-limited settings can be translated into improved health outcomes.

## Introduction: linking more effective research to better healthcare, policy and health outcomes

Gretchen L. Birbeck

**Figure:**
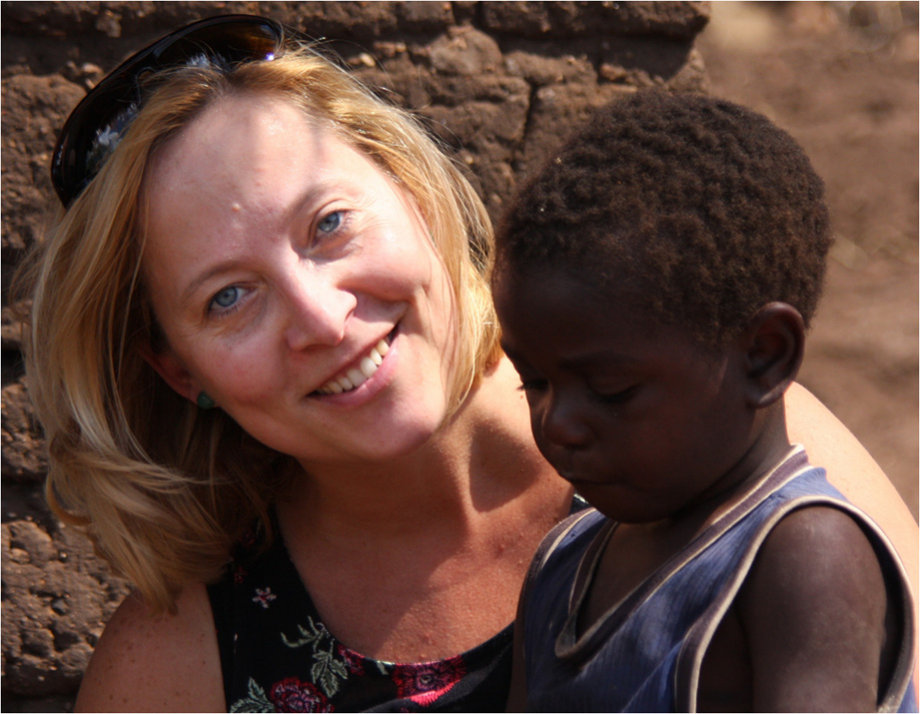
**Gretchen L. Birbeck is a Professor at the University of Rochester and adjunct faculty at the University of Zambia.** With residences in both Zambia and the US, she conducts research, provides clinical services and trains the next generation of clinical researchers on both continents. She has been recognized as a US Paul Rogers Society Global Health Research Ambassador and a World Health Organization Ambassador for Epilepsy. She was a National Finalist for the C. Peter Magrath University/Community Engagement Award for her two decades of work as the Epilepsy Care Team Director at Chikankata Hospital in rural Zambia.

This Medicine for Global Health forum article addressing the who, what, where, when, and so what of evidence-based medicine in resource-limited settings (RLS) includes a collection of commentaries by renowned people from a broad range of geographic settings and a wide array of disciplines. Understanding the drivers of success (and also failure) of different aspects of global health can aid the advancement of better health outcomes.

Charles Shey Wiysonge (Stellenbosch University, South Africa) calls for increased research and research capacity from local researchers in RLS. Although the volume of research on health issues in these regions of the globe has increased exponentially in the last decade, much of the research agenda is still determined by western funding agencies and conducted by western researchers. More leadership by local investigators would better assure that local health priorities are addressed in research and the findings of the work are effectively communicated to policy makers so evidence can be positioned to make a difference.

Edward Mills, a Canada Research Chair for Global Health at the University of Ottawa, points out that unfortunately, where resources are limited, healthcare providers tend to be overwhelmed and under-funded. As a result, one substantial barrier to local research leadership in RLS is the reality that the same overburdened medical personnel who are trying to provide healthcare for the masses are needed to lead research endeavors—usually with inadequate funding, very limited research training, and insufficient local expertise in biostatistics and clinical research methodologies (not to mention no time!). He suggests that the tenuous balance between service provision and the conduct of research could be partially addressed by developing a new cadre of researcher and by local research capacity building in biostatistics and clinical methods.

What follows are excellent examples of what can happen when the right questions are addressed and action taken. Julio Frenk (Dean of the Harvard School of Public Health and former Minister of Health of Mexico) explains how epidemiologic evidence from the Global Burden of Disease work showed Mexico’s protracted epidemiological transition with an impending tsunami of non-communicable diseases (NCDs) in the setting of gross underfunding for healthcare. Major healthcare reforms followed which included universal healthcare and a legal mandate that the healthcare package provided must be reviewed and updated regularly based upon epidemiologic evidence and resource availability.

Mexico is not alone in its progress. Xiao-Nong Zhou, Director of the National Institute of Parasitic Disorders at the Chinese Center for Disease Control and Prevention, reviews the substantial advances China has made toward the Millennium Development Goals. Poverty levels are down and health status measures are up, but the news is not all good. Emerging data indicate that the burden of parasitic diseases may be shifting and expanding as the planet heats up.

Finally, in his inspiring contribution, Prabhat Jha of the University of Toronto reminds us that modest resources well-directed can have major health effects. His compelling story of India’s Million Death Study, funded through a collection of small grants largely targeted at quantifying tobacco-related health hazards, ultimately provided evidence that stimulated action and policy changes related to a diverse range of health-related issues, including 4 to 12 million ‘missing girls’ from gender selective abortions, a previously unappreciated burden of adult malaria deaths and unrecognized HIV/AIDS healthcare needs. Professor Jha credits transparency and open source technology for facilitating data dissemination and appropriate responses by those directing policy and legal structures needed to respond to the evidence.

Universal healthcare in Mexico, poverty reduction associated with improvements in population health in China, modestly funded projects with major public health findings and subsequent responses from policy-makers in India—these examples illustrate that what is happening in RLS today offers lessons for researchers and policy-makers the world over.

### Competing interests

GLB has no competing interests to declare.

## Research for health in Africa - time to move on

Charles Shey Wiysonge

**Figure:**
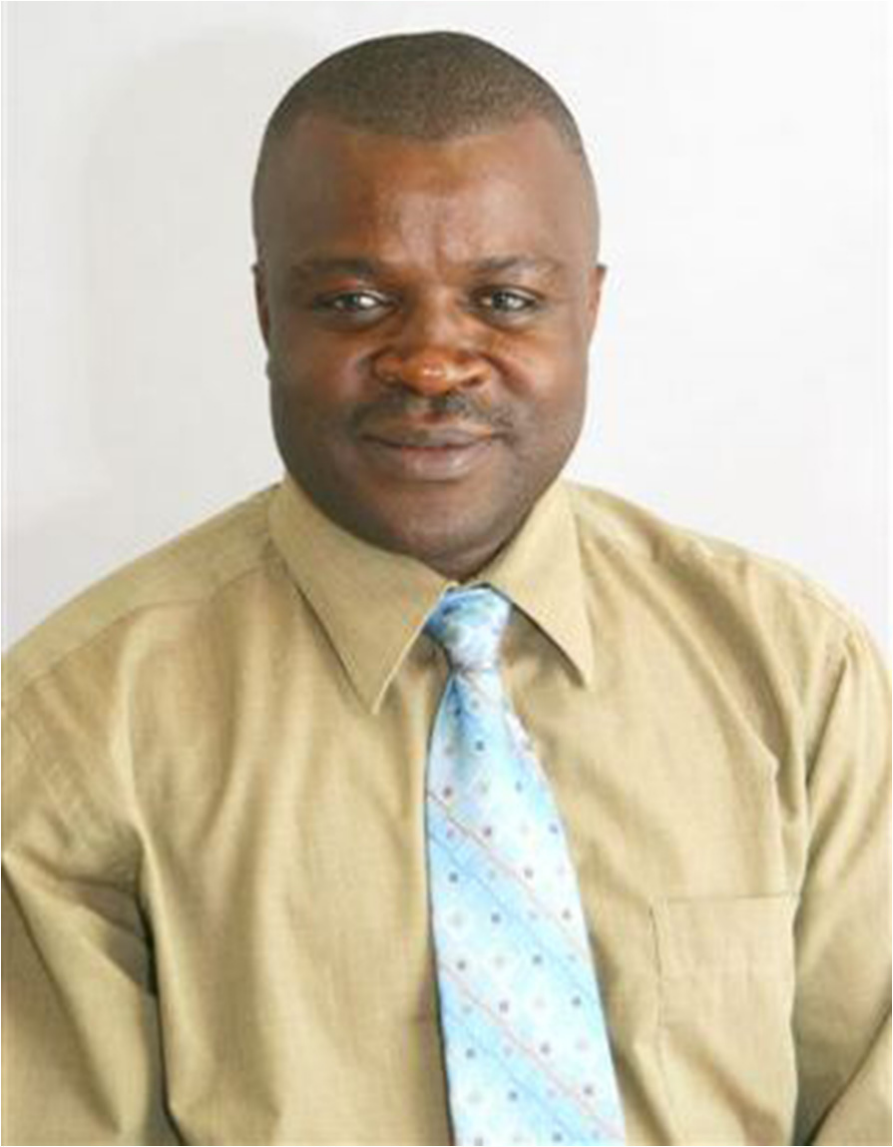
**Charles Shey Wiysonge is the Deputy Director of the Centre for Evidence-based Health Care at Stellenbosch University.** He is a specialist in evidence-based medicine and vaccinology. Before moving into research, Dr. Wiysonge held senior positions in Cameroon’s Ministry of Public Health. His research is united by a set of questions regarding strategies for promoting and protecting health and preventing disease and death in Africa as well as an engagement with systematic reviews and knowledge translation. Dr. Wiysonge was decorated by the Presidency of the Republic of Cameroon in 2011 with the National Order of Valour (the highest honor in Cameroon), in the category ‘Chevalier.’

Research for health (or health research) denotes the creation of knowledge that can be used to promote, restore, maintain, protect, or monitor the health of human populations [1]. Health research seeks to answer questions concerning health, produce evidence required to guide policy and practice, and identify new healthcare interventions [2]. Contextualized health research is critical to ensure healthcare effectiveness, efficiency, and equity in Africa [3]. Research publications have an essential role in the scientific process, providing a strategic connection between knowledge generation and its translation into policy and practice [4]. It is, therefore, of concern that research publication output capacity of African researchers is disproportionately lower than that of their western counterparts [4,5].

The relatively low research productivity in Africa is multifactorial in origin [6]. Important contributors to this problem include the non-alignment of in-country research to national research policies and the nonexistence of national research policies with well-defined priorities [7-9]. The health research field in Africa seems to be characterized by numerous players, varied interests, scattered efforts, and uncertain outcomes in relation to impact on the major health challenges of the continent [4,5]. Organizations based in the North sponsor most of the research reported from Africa [5,8]. In addition, many African countries do not have norms and standards for developing collaborative research agreements with external institutions [7,8]. Without such norms and standards, it is likely that research priorities will be influenced more by the agendas of foreign institutions than by the health requirements and concerns of host countries. Thus, although North–South collaboration is desirable, it is important to question the national ownership of research conducted in African countries under such circumstances. National ownership is essential, as it helps to ensure that national assets are used judiciously and that the host population benefits from research.

We recently conducted a bibliometric analysis of childhood immunization research from Africa and found that research productivity on the continent is highly skewed, and that African researchers make only a minimal contribution to global research output. In addition, we found no significant association between immunization research productivity and immunization coverage (incidence rate ratio 0.38, 95% confidence intervals 0.04 to 3.42) [4]. We attributed the lack of association between increased research productivity and improved service delivery to the lack of sharing of research evidence between researchers and policymakers for translation into policy and public health action.

Africa is a large and complex continent and, despite some countries already having strong research profiles [4,5], optimal growth and efficiency overall can only be realized if the way forward is charted in a systematic and coordinated manner. Increased political commitment to health research by African governments and their development partners would be indispensable for such growth. Political commitment can best be accomplished by defining a clear and considered plan for Africa’s future research enterprise, incorporating a robust accountability framework. The research strategic plan should offer due credit to systematic reviews and knowledge translation [10]. Systematic reviews are summaries of existing research in which bias and chance have been minimized by a systematic identification, critical appraisal, and synthesis of all relevant studies on a specific topic according to a transparent and predetermined process [11]. Without systematic reviews, limited research resources on the African continent will continue to be wasted on unnecessary research and needless confusion will persist from failure to interpret the results of new research in the context of other relevant research [12]. Knowledge translation encompasses all mechanisms for facilitating the uptake of research evidence into policy and practice [13,14]. The research strategic plan and ensuing accountability framework should be used both politically and technically at continental and national levels to bring greater policy consistency and advocate for better action from governments and development partners, in order to increase research output in Africa and spread the full benefit of research to all sectors of the African society by 2015 and beyond.

### Acknowledgements

CSW is grateful to Prof Jimmy Volmink for critical reading and discussion.

### Competing interests

CSW has no competing interests to declare.

## Balancing research with healthcare in resource-limited settings

Edward J. Mills

**Figure:**
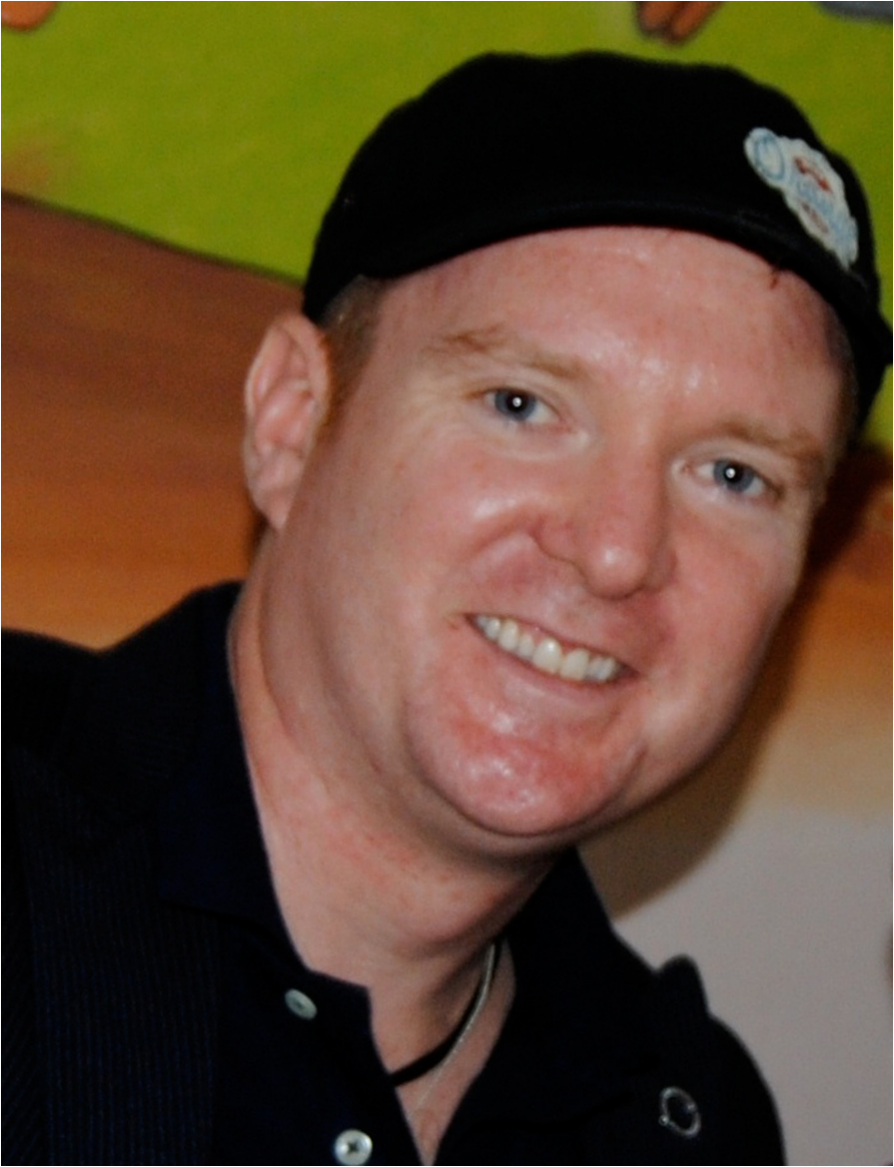
**Edward Mills is Canada Research Chair in Global Health at the University of Ottawa.** He is adjunct faculty at the National University of Rwanda and Stanford University. He is trained in both Clinical Epidemiology and International Law. He works predominantly on issues of HIV/AIDS in Africa. In addition, he runs a statistical methods group that conducts clinical trials and meta-analysis.

RLS are also the environments in which undertreated diseases thrive and guidance on how to treat those conditions effectively is limited [15]. For these reasons, there is both an enthusiasm for conducting locally relevant randomized clinical trials (RCTs) and a need to find out what works in a simple and cost-effective way. Despite this enthusiasm, there are several important impediments that make the widespread conduct of RCTs in most resource-limited settings challenging. These can be impediments conferred on poor settings by outside organizations as well as internal domestic challenges. Herein, I outline what I believe are the three most important challenges.

First, as with almost all poor settings, human resources for health are consistently inadequate for delivering healthcare. There is a constant push-pull influence of research on healthcare as most organizations want to be seen to do research but do not have the requisite staff to conduct quality research. In many circumstances, physicians and clinical officers are prioritized for research training that inevitably takes them away from seeing patients. However, unless the research is paid for by an international organization, physicians and health staff may lose income by conducting research rather than caring for patients. There is a clear need to prioritize individuals for research methods training and a specific cadre of health researchers may be a strategy to increase specialty skills while minimizing the negative effects on the health system. In the absence of paid and allotted time for an individual to conduct research, it is not realistic for an effective clinical research environment to thrive.

Second, there is a specific need for training in biostatistics and clinical research methodology. In my experience, there are many excellent health workers, typically physicians, involved in clinical research projects. However, almost consistently absent are biostatisticians and methodologists. This is important for two reasons: one is to provide input to the design and interpretation of externally funded and designed research; and two is to be able to conduct the analysis at a local level regardless of external collaboration. Some international organizations, such as the International Clinical Epidemiology Network (INCLEN), have recognized this [16] and made important efforts to build capacity by partnering organizations and mentors. However, this needs to occur with every country that has an active research agenda and prioritizing high-level biostatistics will require an investment by domestic ministries of health. This is a particular challenge in most settings as the highest qualified biostatisticians and methodologists are frequently recruited by international organizations that can pay much better salaries than domestic ministries.

Third, there is the need for freedom of decision-making for researchers. This is a particularly nuanced topic as every culture is different. Because health typically has a hierarchy of decision-making power, often led by the most senior physicians and then subsequent cadres of workers, the freedom for a junior researcher to critique common practice is limited. In many cultures, questioning authority is considered rude. Similarly, the research agenda may already be set by an external funder. For example, U.S. Agency for International Development (USAID) supported organizations may be mandated to meet USAID needs. Innovative strategies to provide small research grants may be one method to identify new and innovative locally-relevant research topics. Organizations such as Grand Challenges Canada and the Micro-research grants project recognize this [17].

Unless these challenges can be directly addressed by both domestic and international agencies and funders, then conducting RCTs will remain the domain of well-funded external collaborators. In the new era of global health education, it will be a shame if we do not build and support the infrastructure for long-term, locally-relevant clinical research.

### Competing interests

EJM has no competing interests to declare.

## Knowledge to transform global health and policy: the success of universal health coverage in Mexico

Julio J. Frenk

**Figure:**
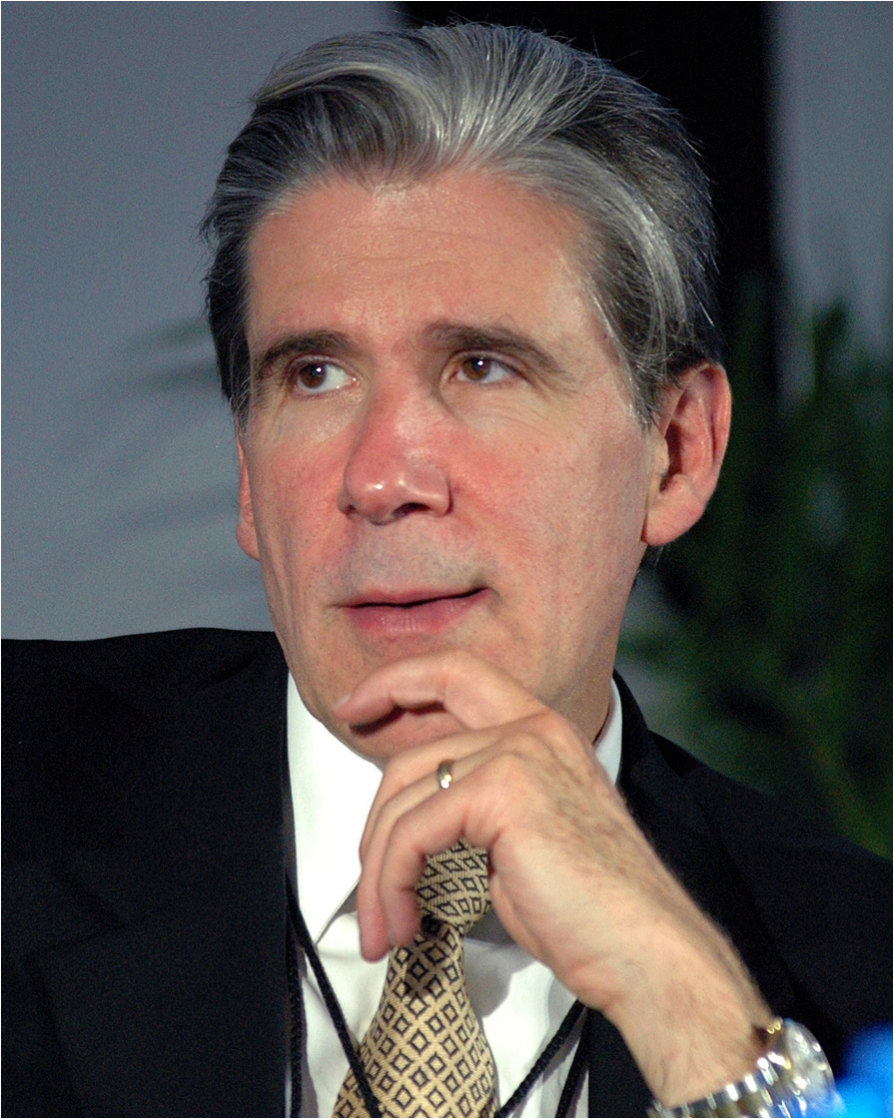
**Julio Frenk is the Dean of the Faculty at Harvard School of Public Health and T & G Angelopoulos Professor of Public Health and International Development, a joint appointment with the Harvard Kennedy School of Government.** Dr. Frenk served as the Minister of Health of Mexico from 2000 to 2006, where he introduced universal health coverage. He was the founding director of the National Institute of Public Health of Mexico and has also held leadership positions at the Mexican Health Foundation, the World Health Organization, and the Bill and Melinda Gates Foundation.

Knowledge is the most powerful force for enlightened social transformation. When it is translated into evidence, it provides a solid foundation for policy design and program implementation.

Before becoming dean of the Harvard School of Public Health, I lived through the fascinating experience of conducting a large-scale reform of a health system. This process reached a major milestone in 2012, when Mexico announced to the world the achievement of universal health coverage (UHC). With more than 50 million previously uninsured persons now covered through a new public insurance scheme, this developing country has reached a globally cherished goal that has nonetheless eluded most poor nations and a notorious rich one.

UHC was reached through a reform that was designed and implemented making use of evidence derived from the local adaptation of knowledge-related global public goods, such as the measurement of global burden of disease, the framework for health system performance assessment and the methods for calculating national health accounts, among others.

Thus, the analysis of the burden of disease showed that Mexico was undergoing a protracted and polarized epidemiological transition characterized by the complex coexistence of an unfinished agenda (common infections, maternal mortality, under-nutrition) with emerging challenges (NCDs, injuries, mental health problems, obesity). The Mexican health system, however, had not kept up with the pressures derived from this transition. At the turn of the century, Mexico was spending only 5.6% of its gross domestic product (GDP) on health, far below the average figure for the Latin American region (6.7%). Even worse, more than half of the total health expenditure was out-of-pocket, which exposed households to major financial shocks. This was a direct result of the fact that approximately half of the population lacked health insurance.

Such evidence generated the advocacy tools to promote a legal reform establishing in 2003 a system of social protection in health which has reorganized and increased public funding by a full percentage of GDP over eight years in order to provide universal health insurance through a new public scheme called Seguro Popular (SP). This insurance scheme guarantees access to a comprehensive package of cost-effective services covering the prevention, early detection, diagnosis, treatment, and palliation of the major causes of ill health, including NCDs. The law stipulates that the package must be progressively expanded and updated annually on the basis of changes in the epidemiologic profile, technological developments and resource availability. The gradual expansion of population and intervention coverage, coupled with demand-side subsidies and supply-side incentives for efficiency, made this a fiscally responsible and sustainable reform—and garnered the crucial support required from the Ministry of Finance.

The reform was also subject to a rigorous external evaluation which showed major impacts. A community trial developed in 2005/2006 demonstrated that SP is expanding access to health services, reducing out-of-pocket expenditures and providing protection against catastrophic health expenditures especially to the poorest households [18].

The Mexican experience shows the importance of mobilizing knowledge in pursuit of socially valued policy objectives. Now the evidence produced by this national reform feeds back into the global pool of experience, thus generating a process of shared learning.

### Competing interests

JJF has no competing interests to declare.

## Reducing the burden of infectious diseases associated with poverty in China

Xiao-Nong Zhou

**Figure:**
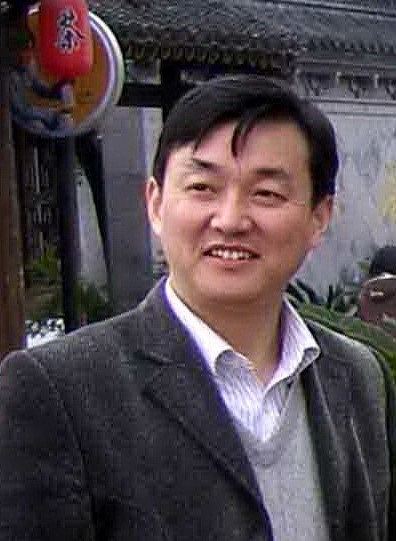
**Xiao-Nong Zhou is Director of the National Institute of Parasitic Diseases at the Chinese Center for Disease Control and Prevention, based in Shanghai, P.R. China.** Currently, Professor Zhou is serving as Chair of the National Expert Advisory Committee on schistosomiasis and other parasitic diseases for China’s National Health and Family Planning Commission (formal Ministry of Health). He has collaborated with WHO/TDR and WHO, for instance he is member of WHO/TDR STAC, member of WHO STAC on NTDs, member of WHO Foodborne Burden Epidemiology Reference Group. He had contributed to the Regional Network on Asian Schistosomiasis and Other Helminth Zoonoses during 2007 to 2012 as former President and to the WHO Thematic Reference Group on Environment, Agriculture and Infectious Diseases during 2009 to 2011 as co-chair.

In order to meet the eight Millennium Development Goals (MDGs) by 2015, more international communities and countries are taking further actions on human development issues. At the same time, they are also seeking the solutions to the post-2015 agenda for sustainable development, since many counties or regions will have difficulty in achieving these goals by the 2015 deadline.

China is one of few developing countries that has made enormous progress towards the achievement of its MDGs. Since 1990, poverty, especially absolute poverty in rural areas, has been greatly reduced, life expectancy has increased to 74.8 years, the maternal mortality ratio has dropped to 26/100,000, and the infant mortality rate is currently 12/1,000 [19,20]. Due to the efforts to control infectious diseases, there has been a significant decline in both the prevalence and disease burden of infectious diseases in China compared to 60 years ago [21,22].

Similar to other developing countries, China is facing challenges in its sustainable human and economic development. First, there is a need to balance regional economic growth with equitable and sustainable development. Second, China’s already stressed environment is experiencing additional stress caused by rapid industrialization, urbanization and the significant increase in individual consumption. These challenges have a negative impact on the quality of public health. For example, certain neglected tropical diseases, such as soil-transmitted helminthiasis and schistosomiasis, affect more than 465 million people in P.R. China [23,24]. These diseases tend to disproportionately affect those living in the remote rural areas who are relatively resource-poor [25]. Therefore, greater efforts are required for China to take further actions on combating these infectious diseases of poverty [26].

One of the ways to tackle infectious diseases of poverty better is to identify research gaps and set priorities towards eliminating these diseases. I was involved in a World Health Organization (WHO) think tank comprising more than 100 experts that aimed to address these issues through various seminars and workshops. In particular, as the co-chair of the WHO Thematic Reference Group on Environment, Agriculture and Infectious Diseases, we made important recommendations with regard to trans-disciplinary research priorities using the one-health approach [27]. These aim to break the cycle of the infectious diseases and poverty, and focus on issues such as environmental changes, innovative technology, social determinants and health systems [28].

In particular, the impact of climate change on the transmission of infectious diseases of poverty within low and middle income countries was recommended as one of the high priorities [29]. A specific example, and one on which my research efforts are focused, is the increased potential transmission of schistosomiasis due to global warming [30]. Based on our biology-driven model, our group found that global warming would result in an increase of transmission of schistosomiasis japonica, due to disease epidemic areas extending northwards into currently non-endemic areas, and transmission intensity increasing during transmission season [31]. Employing time-series modelling and geostatistical analysis of temperature records supported by geographic information system (GIS) and remote sensing (RS) technology, we estimated that a surface area of 41,335 km^2^ could become potential schistosome-transmission areas, putting an additional 21 million people at risk for an infection with *Schistosoma japonicum*[32]. No doubt such a result has a profound public health impact and is of considerable economic significance. Our field studies also illustrated that climate change contributes to an increased frequency of extreme climate events, resulting in increased rainfall and widespread flooding which caused the resurgence of schistosomiasis along the Yangtze River after the big flood of 1998 [33]. Therefore, it is worthwhile to apply an integrated tool employing a GIS/RS approach and modeling methods in the prediction of the potential distribution areas of schistosomiasis under global warming scenarios [34].

### Competing interests

XNZ has no competing interests to declare.

## Transparency in counting the dead and describing causes

Prabhat Jha

**Figure:**
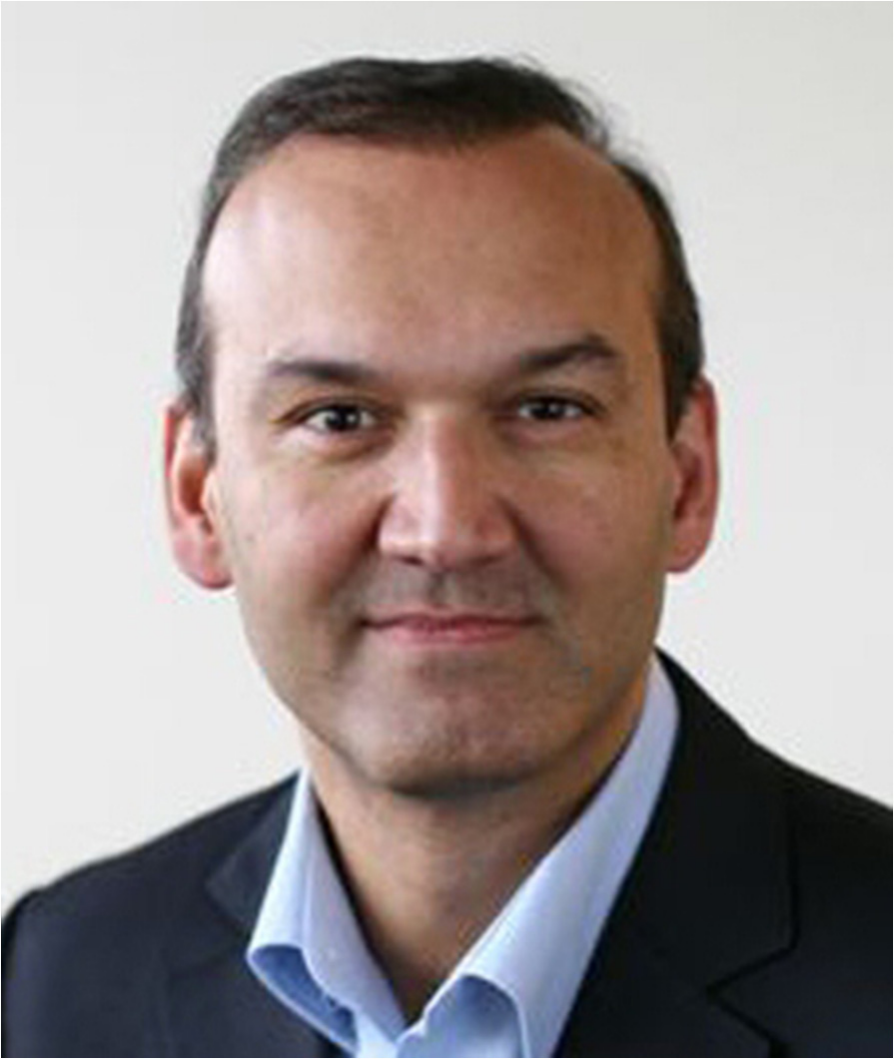
**Prabhat Jha is the University of Toronto Endowed Professor in Disease Control at the Dalla Lana School of Public Health, and the founding Executive Director of the Centre for Global Health Research at St. Michael’s Hospital.** He is the lead investigator of the Million Death Study in India, which quantifies the causes of premature mortality and the contribution of key risk factors, such as tobacco and alcohol.

Prior to 2004, it had been difficult to assess accurately what killed Indians since India is a country similar to most other low and middle-income countries where death registration by cause remains uncommon and most deaths occur at home without medical attention. To address the fundamental gap, the Million Death Study (MDS) was launched in 2004. Led by the Registrar General of India (RGI), the MDS has employed a simple, practical and action-oriented approach to survey the causes of death from an enhanced verbal autopsy [35,36]. Among the findings thus far:

● HIV/AIDS resulted in 0.1 million deaths (UNAIDS estimated 0.4 million [37]) in 2004; this result led to adjustments in AIDS funding to align better with actual demand for life-prolonging therapies.

● Smoking caused approximately one million deaths [38] in 2010; this finding led India’s government to introduce warning labels on cigarette packages and raise tobacco taxes to help reduce consumption [39].

● Malaria caused 0.2 million deaths (13 times the WHO estimate [40]) in 2005 primarily among adults; within one week, this finding led to public demand for greater control of malaria in the state of Orissa (and spurred other, currently inconclusive, research on adult malaria deaths in Africa).

● Selective abortion of females accounts for about 4 to 12 million ‘missing girls’, with about half of these just in the last decade [41]; within 10 days of publication, this finding prompted stricter rules on ultrasound testing/reporting.

● Two million child deaths occurred in 2005 (down to 1.5 million in 2012) from five avoidable causes [42]; this finding spurred the expansion of neonatal/intra-partum care and is presently enabling district-based monitoring of child deaths and up-to-date estimates of child mortality by gender. Only about 80 districts comprise nearly one third of India’s child deaths [43], while gender disparities are far more widespread. This has enabled the ‘district report cards’ to try to accelerate child mortality declines, focused on specific introduction of new vaccines, expanded case management and strategies to reduce neonatal deaths [44].

Many of these findings were not predicted in the initial study design (which was mostly focused on quantifying tobacco hazards among adults and was funded by three small National Institutes of Health and Gates Foundation tobacco research grants).

Transparency in these data is important as it helps build confidence of decision makers and funders in the results. The field work, training methods and coding practices in the MDS are all open source (and freely available at http://www.cghr.org). This has spurred replication, which is welcome. We are in active discussion with the RGI to make the primary data openly available to all researchers to use imaginatively and without restriction. Unfortunately, too many datasets, including the Global Burden of Disease remain closed, and hence the basic scientific standard of reproducibility limits their scientific and public credibility. It takes time to change government policies based on scientific findings, but I am confident that India’s tradition of open debate and democratic institutions will lead to data improving public health.

India gave the world the zero, and now it is showing the world how reliable, low-cost cause of death data can transform health strategies.

### Competing interests

PJ has no competing interests to declare.
